# Evaluation and Risk Assessment of Congenital Anomalies in Neonates

**DOI:** 10.3390/children8100862

**Published:** 2021-09-28

**Authors:** Rita P. Verma

**Affiliations:** Department of Pediatrics, Division of Neonatology, Nassau University Medical Center, East Meadow, NY 11554, USA; rverma2@numc.edu or ritaverma@aol.com; Tel.: +1-516-5723319 or +1-6318755375

**Keywords:** congenital anomalies, risk assessment, genetics, phenotype, infant mortality, morphogenesis

## Abstract

Congenital anomalies (CA) are a large heterogeneous group of disorders of abnormal morphogenesis or biochemistry which present at birth and carry widely variable implications for morbidity and mortality. They are the leading cause of infant mortality in the USA, with an incidence of 3–4% of all births. CA are the fourth leading cause of neonatal mortality worldwide, with an estimated 295,000 deaths annually. The enormous variability in the clinical presentation in terms of severity, time of occurrence, course, complications, management, and outcomes makes the evaluation of CA complicated, highly specific, and individualized. The anomalies can impart tremendous physical, social, and emotional distress on the patient with massive emotional, social, financial, and medical implications for the family and society. The diagnosis may remain elusive despite rigorous, elaborate, and extensive investigations in many cases. While the enormous strides in genetic testing and gene modification therapy have an encouraging impact on the diagnosis and treatment, the risk assessment of recurrence in the family and population of CA remains obscure in most cases due to the lack of information and referable evidence.

## 1. Introduction

Congenital anomalies (CA) are the leading cause of infant mortality in the USA and account for an estimated 20.3% of all deaths during infancy [1]. With an incidence of 3–4% of all births, they are the fourth leading cause of neonatal mortality worldwide, with 295,000 deaths reported annually [2,3]. Congenital malformations form a heterogeneousgroup of morphological, functional, or biochemical defects that can manifest in utero, at birth, or during the post-natal period with widely variable and unrelated etiopathogenesis, clinical profile, and management principles. The sign and symptoms may range from being mild, moderate, and severe to lethal. The deformity may be devastatingly disfiguring or merely cosmetic. Death may occur antenatally, at or soon after birth, during the neonatal period, infancy, or later in life. The etiological factors are as variable, being genetic, environmental due to exposure to teratogenic agents, infectious, or socioeconomic and demographic factors, including maternal nutrition status. The personal, familial, social, medical, and financial impact may be minimal or staggering depending on diagnosis and classification.

The five most common birth malformations in the USA are club foot, Down syndrome, pulmonic stenosis/atresia, cleft palate, and limb deformities while, congenital heart disorders, neural tube defects, and Down syndrome constitute the three commonest causes of mortality due to CA in that order. As for economic implications, 35% of the hospitalization cost of the pediatrics population is attributed to CA in the USA, and the overall annual cost of hospitalization due to congenital malformations is USD 22.9 billion [4].

The striking heterogeneity in almost all aspects of CA and the enormity of social, familial, and financial impacts make the evaluation and management baffling and highly specialized, especially in regard to the risk assessment for recurrence, which is an integral part of the management. This review provides a comprehensive overview of CA in children with a specific section on the recurrence risk of inheritable disorders

## 2. Classification and Etiopathogenesis

A congenital anomaly is an outcome of abnormal morphogenesis. Normal morphogenesis is a systematic process that involves a combination of several simultaneously or sequentially occurring histo-physiological activities at the cellular and molecular levels, namely cell migration, aggregation of identical cell types, the interaction between neighboring tissues, controlled cell death, mechanical forces, and hormonal effects. Any disruption in these processes may result in a structural, biochemical, or functional anomaly. CA may occur as anomalous phenotype, genotype, or both. While phenotype defines morphologic and functional attributes of an individual’s organs, tissues, or cells, genotype denotes the genetic makeup. Genotype is inherited from parents or may occur sporadically as a de novo mutation. The phenotype may be affected by genotype and epigenetic modifications, and environmental factors, including lifestyle.

Pathologically, CA can be categorized into those caused by genetic or nongenetic factors [5]. Some anomalies are etiologically multifactorial and triggered by a combination of genetic and environmental reasons, while approximately 50% of the cases remain idiopathic. The genetic and nongenetic groups are further divided into several sub-categories. Basically, genetic abnormalities may be due to chromosomal or single-gene (monogenic) disorders, which have autosomal recessive or dominant, and X-linked modes of inheritance. An epigenetic phenomenon called genomic imprinting has been detected in rare disorders in which genes are expressed in a parent- of- origin-specific manner. The epigenetic marks are “imprinted” in the sperm or egg germlines of the parents and are maintained through mitosis to pass over to the somatic cells of the offspring. While paternal inheritance of deletion at band 11 of the long arm of chromosome 15 results in Prader–Willi syndrome with hypotonia obesity and hypogonadism, maternal inheritance of the same deletion expresses as Angelman syndrome characterized by epilepsy, tremors, and a smiling facial expression. Other conditions consequent to this phenomenon are Beckwith–Wiedemann syndrome, Silver–Russell syndrome, pseudohypoparathyroidism, and transient neonatal diabetes mellitus. In uniparental disomy, both members of a chromosomal pair are transmitted from one parent, allowing a recessive gene to express. In inheritance via allelic expansion, a gene segment expands in size when transmitted from parent to child and causes specific disease, one example being fragile X syndrome. Nongenetic and teratogenic etiologies of CA consist of the following: maternal disorders, such as phenylketonuria, infection, and diabetes mellitus; in utero teratogens’ exposure; intrauterine accidents such as amniotic bands formation leading to amputation; in utero space constraints due to multiple gestations, uterine anomalies, or oligohydramnios resulting in deformation; and congenital infections. Examples of some of the multifactorial disorders caused by the interactions of various genes and environmental factors are non-syndromic cleft lip/palate, congenital heart diseases, and neural tube defects. About 4–6% of all CA are supposed to be due to teratogenic influences.

Based on the clinical implications, the anomalies are classified as major and minor. While minor malformations may be of cosmetic significance only, major anomalies are widely variable in severity, in some cases being life-threatening, even lethal. According to the population studied, the prevalence of major malformations is reported to range from 1 to 4 percent, whereas that in minor anomalies may be as high as 35% in certain populations—the discrepancies underscoring the importance of environmental and regional influences [5]. The presence of three or more minor anomalies indicates significant defects in morphogenesis. Even though detected in only 0.5% of births, almost 90% of such cases have >1 major anomaly, which might eventually fall into the diagnosis of associations or syndromes. Major anomalies are generally due to molecular defects that interfere with normal morphogenesis processes like apoptosis, intracellular signaling, migration of neural crest derivatives, and chromatin remodeling. Some of the genes (e.g., Homeobox genes in Synpolydactyly, microphthalmia, and holoprosencephaly), transcription factors (e.g., deletions of T-box 1 in conotruncal heart defects of DiGeorge syndrome), fibroblast growth factor receptors (in craniosynostoses syndromes), and enzyme defects (such as cholesterol biosynthesis leading to Smith–Lemli–Opitz syndrome) have been identified as etiopathogenic factors for specific major malformations.

## 3. Anomalies of Phenotype

Abnormal morphogenesis may manifest in a variety of ways at the macroscopic and microscopic levels [6]. Some of the common patterns and terminologies used in the description of abnormal phenotypes are as follows.

**Figure 1:**
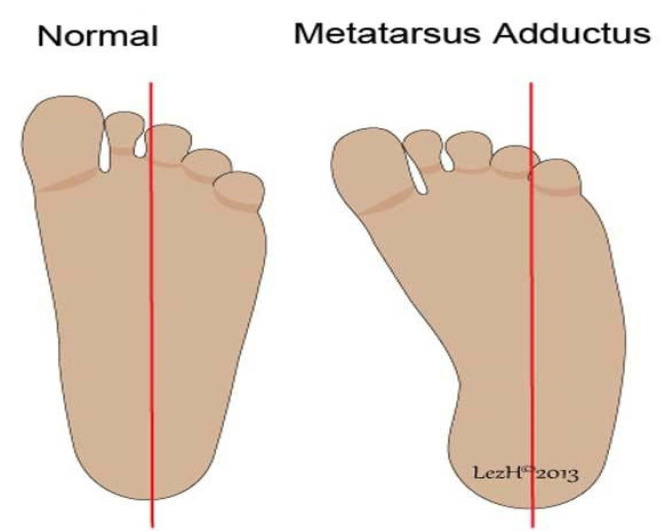
Metatarsus adductus as an example of deformation due to intrauterine positioning or space constraints.

**Figure 2:**
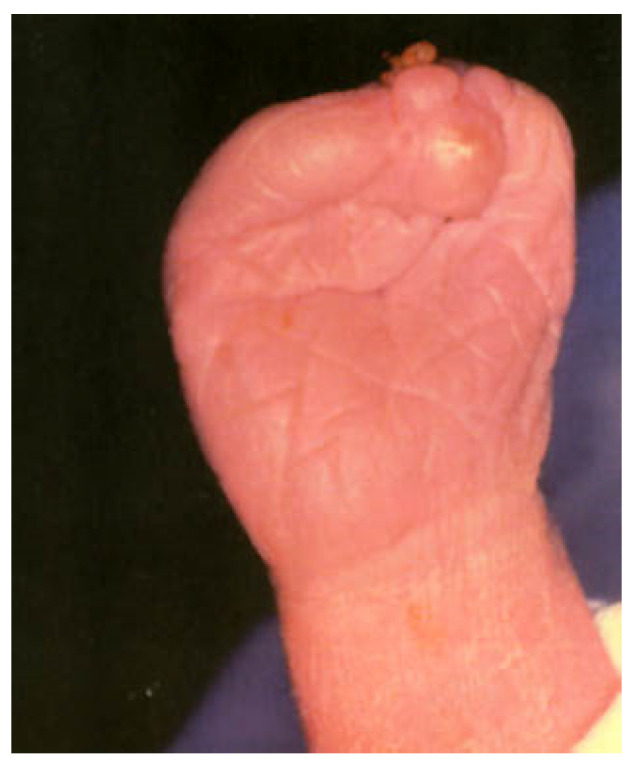
Amputation of digits with a constricting amniotic band as an example of disruption.

1. Anomaly is defined as a significant morphological or anatomic variation in the phenotype from the standard reference population. They may be macroscopic or microscopic and are categorized into major and minor. Major anomalies have significant adverse implications in functional, anatomical, psychological, social, or cosmetic wellbeing, and minor anomalies are variants with no significant medical or major cosmetic consequences.
2. The malformation is a non-progressive morphologic anomaly brought about primarily by an intrinsic error in the developmental process at the cellular or molecular levels of an organ or a body part. Malformations may be isolated or part of a syndrome and are causally heterogeneous, brought about by gene mutations, exposure to teratogens, or a combination of both. For example, the limb anomaly in Holt–Oram syndrome is genetic, while it is due to teratogenic exposure in thalidomide embryopathy.
3. Deformation refers to a distortion of the shape or size in an otherwise normally developing or developed body part of a fetus and is caused by aberrant extrinsic or intrinsic mechanical forces. Deformations are causally heterogeneous and lead to the loss of alignment or abnormal positioning and distorted configuration. Uterine space constraints due to oligohydramnios, multiple gestations, and uterine anomalies may result in craniofacial asymmetry, arthrogryposis, and metatarsus adductus (Figure 1). Deformation generally occurs after organogenesis, but it may permanently alter the structural relationships if mechanical transduction happens during embryogenesis. Deformations may or may not be reversible.
4. Disruption is defined as an inherently static morphologic abnormality brought about in utero by some destructive mechanical forces acting upon an otherwise normally developing or developed fetal tissue or physical part. It results in the destruction of the involved body part with cell death and may cause the developmental arrest of the adjacent tissues, thereby leading to a secondary malformation (Figure 2). It can be an initial event in a sequence of events if it occurs early in gestation. It is causally heterogeneous and may be isolated or part of a syndrome or other broader patterns. The mechanisms of cell death and tissue disruption include vascular compromise, anoxia, teratogens exposure, infections, or mechanical forces. Missing digits or limbs are examples of disruption. The process characteristically affects several tissue types in a specific anatomical region, and the phenotypic abnormalities may cross multiple embryonic cell lines. Some pathological developmental processes can cause both disruption and deformation. For example, constriction rings at the tip of a finger leading to swelling associated with fibrous strands of tissue resulting in loss of digits. Amniotic bands encircling a limb are one potential reason for the loss and disruption of an extremity.
5. Dysplasia is an abnormality in the growth and development of cell or tissue histology, which leads to abnormal anatomical structure or physiological function resulting from the growth. Dysplasias are causally heterogeneous and can be triggered by genetic factors, teratogens exposure, or metabolic disorders. It can be widespread or confined to a single organ. It may involve single or multiple germ layers and single or multiple organs. It may be very diverse, localized or generalized; unilateral or bilateral; focal or multifocal; benign, malignant or premalignant; static or progressive; or evanescent. It can happen at the cellular level (microscopic), examples being bronchopulmonary dysplasia and fibrous epithelial dysplasia, or in organs (macroscopic) such as renal dysplasia. It may be a dynamic or an ongoing process and can begin prenatally or postnatally. Dysplasia may be associated with malformations.
6. Sequence refers to the phenomenon of a single or multiple morphological anomalies cascading from a single primary anomaly, which might be malformation, disruption, dysplasia, or deformation. The resultant anomaly is not an essential and direct derivation of the primary cause, as in genetic abnormalities. A sequence may be an isolated phenomenon or a component of a pattern or syndromes and is causally heterogeneous. For example, in the Pierre Robin sequence, a small mandible, the primary anatomical defect, leads to protruding tongue, which may interfere with the palatal closure and create a cleft palate. Sometimes it may be difficult to distinguish between the primary and the consequential effects.
7. Association is defined as a pattern of morphological anomalies which are not causally related but occur together more often than would be expected by chance only. Some associations may be syndromes with overlapping features. Such cases may eventually be identified to have a pathological etiology and then moved to the category of a syndrome. For example, the CHARGE association, after identification of the causative gene, is now identified as CHARGE syndrome.
8. A syndrome is a combination of causally but not necessarily pathogenically related anomalies that are characterized into a specific condition. The anomalies can be malformations, deformations, disruptions, sequences or dysplasia, major or minor, or functional, such as those affecting the neurological, cognitive, sensory, or behavioral performances.

## 4. Anomalies of Genotype

Inheritable and genetically determined congenital malformations can occur due to chromosomal or single-gene (Mendelian) defects. Single gene disorders (SGD) have autosomal and X-linked dominant and recessive patterns of inheritance. Cystic fibrosis is the commonest SGD reported in the Caucasian population. Inherited disorders may also be caused by defects in the mitochondrial chromosomes [7]. This category has the highest phenotypic variability due to heteroplasmy and the fact that mitochondrial DNA has an increased incidence of mutation [8]. As the mitochondrial DNA is inherited from the mother, the genetic transmission occurs from the affected mother only. Examples are neonatal lactic acidosis, eye anomalies, and Leigh syndrome. Another group of disorders with abnormal genotype may have multifactorial etiopathogenesis with both genetic and environmental components.

**Figure 3:**
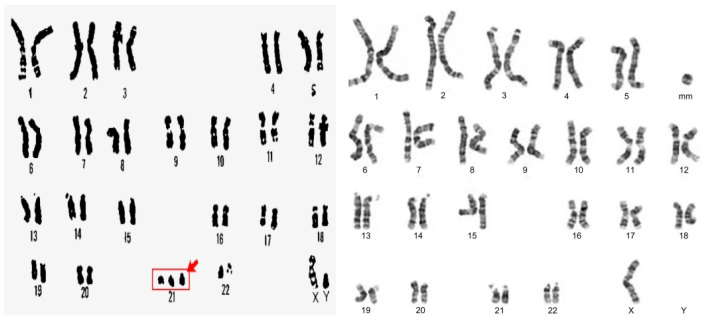
Examples of aneuploidy with Trisomy 21 showing an extra chromosome 21 (**left**) and Turner’s syndrome with missing genes from the short arm of the X chromosome (**right**).

Chromosomal anomalies may result from numerical aberrations (maldistribution) or structural anomalies (rearrangements) of chromosomes. A numerical error in the array of chromosomes is termed aneuploidy, which presents as polyploidy with addition or monosomy with a reduction in the number of chromosomes (Figure 3). Most of these conditions result from the failure of chromosomes to disjoin during meiosis (non-disjunction). Some of the common examples of aneuploidy are the trisomy syndromes or Turner and Klinefelter syndromes.

**Figure 4:**
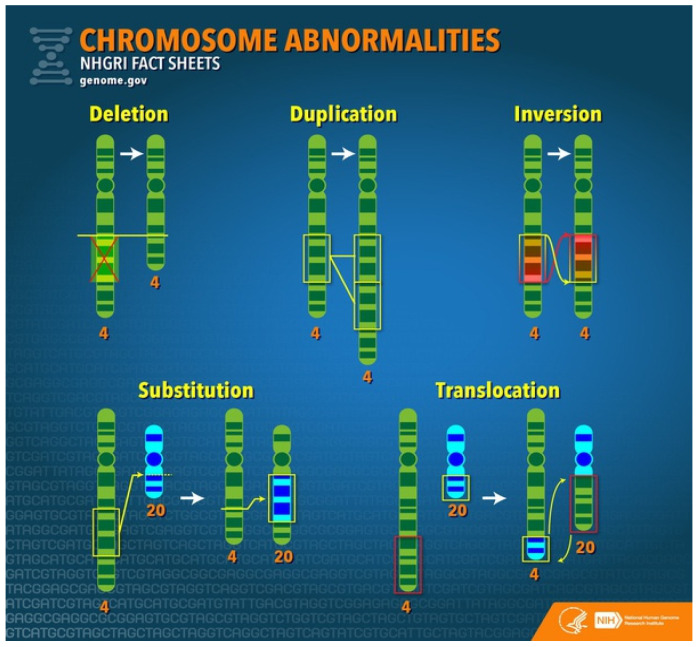
Common structural chromosomal anomalies resulting in abnormal genotype.

Structural chromosome disorders result from the breakage and rearrangement of segments or parts of a chromosome (Figure 4). Some of the common anomalies are described below.

Deletion refers to the loss of a piece or section of chromosomal material. If too small to be visualized under a microscope, it is termed microdeletion. Deletions can be terminal if only one break is present at the end or interstitial if two pieces of chromosome material are lost from within the chromosome. There is only one copy of a particular chromosome segment instead of the usual two copies in deletion syndromes.

Duplication presents with an extra copy of a segment of a chromosome. So there are three copies of a particular chromosome segment instead of the usual two.

Mutation indicates a change in the DNA sequence that leads to a change in its function. A mutation may be silent (no overt clinical signs or symptoms as amino acids may be encoded on different codons); missense (a new nucleotide changes the codon); nonsense (new nucleotide changes the codon to a STOP codon so that the mRNA translation is stopped) or splice-site when mutation at splice site prevents removal of an intron.

Translocation happens when genetic material is exchanged between two chromosomes. It may be balanced with no gain or loss of DNA and no anomalies or unbalanced when the process results in gain or loss of chromosomal material. Even though there may not be any phenotypical anomalies, balanced translocations might have implications for the patient’s offspring.

Reciprocal translocation is an anomaly in which the phenotype is normal despite the break and exchange of material between two chromosomes.

Robertsonian translocation is another example in which the phenotype may be normal. It involves only selected chromosomes, such as 13,14,15,21,22. In this anomaly, the short arm is lost, and long arms fuse at the centromere.

Inversion is described when a piece of a chromosome breaks at two places and then is reinserted in the opposite direction. Inversions may be paracentric if it does not involve centromere or pericentric if the centromere is involved.

Isochromosomes result from abnormal mitosis in which a break at centromere results in two short (p) arms or two long (q) arms from the same side, both arms carrying identical genes.

Dicentric chromosomes are defined as the abnormal fusion of two chromosome pieces, both having a centromere.

Ring chromosomes form when the deletion happens at the ends of both arms of the same chromosome, and the remaining chromosome joins, making a ring-like shape. The chromosomes may be eventually lost, resulting in monosomy.

## 5. Evaluation of an Infant with Congenital Malformations

The clinical assessment of a child born with an anomaly begins with a comprehensive history and detailed physical examination and should encompass the following steps.

A comprehensive history of parents, with special attention to ethnicity, age, gravidity, parity, miscarriages, stillbirth, pregnancy-related complications, history of prescription drugs intake, professional or other exposure to toxic agents, illicit drug and other substance abuse, fever or significant illnesses, vaccination status and consanguinity among others.

A detailed history of extended family and pedigree, up to four generations if possible, to evaluate genetic causes.

A thorough physical examination, which should consist of a detailed assessment of craniofacial profile for dysmorphology and of individual organ systems, including the vertebral column, extremities, and skin.

The histopathological features of the placenta and umbilical cord.

The performance of specific diagnostic tests selected on the basis of the results of history and physical examination and individualized according to the case.

## 6. Physical Examination

A detailed systematic examination is an invaluable and integral part of the assessment of a child born with anomalies. It should be standardized and performed by a trained dysmorphologist [9]. Referral to a geneticist is suggested if one major or more than two minor anomalies are present.

The following is a list of specific features and characteristics to look for during the general evaluation of an infant with congenital malformations.

1. Growth parameters: The physical examination starts with the standard measurements of weight, length, and head circumference to establish growth status (small or large for weight) and microcephaly or macrocephaly. In infants suffering from short stature, skeletal dysplasia, or suspected Marfan syndrome, arm span and lower segment/upper segment ratio are useful variables to note.
2. Craniofacial and Cervical assessment: microcephaly; macrocephaly; plagiocephaly; brachycephaly; midface hypoplasia; prognathism, retrognathia, micrognathia; facial asymmetry; hypertelorism, hypotelorism; ophthalmoplegia; esotropia, exotropia; cataract; nystagmus; ptosis; inner canthal distance, outer canthal distance, interpupillary distance; palpebral fissures length; long or short, anteriorly or posteriorly rotated ears; low set ears; pinna length; microtia; prominent, bulbous nasal tip; slit appearance of nose; anteverted nares; long or smooth philtrum; macrostomia, microstomia, high arched palate, cleft lip/palate; cleft uvula; macroglossia, protruding tongue; wide or short neck, neck webbing.
3. Skin, Limbs, chest and Vertebral column: pectus excavatum or pectus carinatum; wide-spaced nipples, kyphosis, scoliosis, spina bifida, deep sacral dimple, sacral hair tuft, sacral tag; sirenomelia; limited range of motion of extremities, contracture; arthrogryposis; polydactyly syndactyly, brachydactyly, arachnodactyly, broad thumbs, and toes; clubfoot; sparse or excess body hair; abnormally light hair; skin hyperpigmentation or hypopigmentation; albinism; nail dystrophy.
4. Genitourinary system: breast development; Tanner staging; the ambiguity of genitalia, penile and clitoris size, micropenis, cryptorchidism, hypoplasia of labia, vaginal hypoplasia/atresia.
5. Cardiorespiratory system: chest asymmetry, chest wall defects, cardiac extrophy, lung sounds, precordial thrill, apical impulse, murmur, arrhythmias.
6. Neurological system: muscle size and tone, any lateralizing or localizing motor or sensory deficits, neonatal reflexes.
7. Abdomen: any mass or tumors, organomegaly, anterior wall anomalies or defects, prune belly, hernias, swelling, scaphoid abdomen.

## 7. Specific Tests and Laboratory Investigations

Massive strides in the knowledge of genetics, epigenetics, and biochemical sciences, as well as bioinformatics, have changed the profile of diagnostic assessment of congenital anomalies. New and innovative tests are available, some centralized in major institutions or laboratories, which should be individualized and selectively utilized on a per case basis. The need and selection of testing are determined by and depend upon the history and physical examination results. The initial specific tests that are commonly performed include computed tomography (CT), and magnetic resonance imaging (MRI) scans of the central nervous system (CNS), echocardiogram, abdominal ultrasound or CT scan, skeletal survey, and autopsy if the patient expires. An ultrasound evaluation of the genitourinary system is indicated in the conditions of ambiguous genitalia to detect the presence of abdominal gonads and uterus and assess the anatomy of kidneys, ureters, bladder, and testicles which are then followed by specific tests. Fundoscopy may be done to detect ocular anomalies, such as retinal colobomas, optic disc defects, and chorioretinitis. TORCH infections and other significant viral infections should be ruled out by performing specific tests as clinically indicated.

## 8. Tests for Suspected Genetic Disorders

The selection of chromosomal studies is guided by clinical presentation (Table 1). Exome sequencing helps in the identification of rare single-gene disorders and is indicated in the conditions of multiple, complex congenital anomalies with no otherwise identified genetic defect. Molecular-based chromosome microarray studies (array comparative genomic hybridization or aCGH) are utilized to diagnose microdeletions. aCGH is the first line of testing in children with multiple anomalies and intellectual disabilities [10]. It has now replaced Giemsa banding karyotype (G-banding) and fluorescent in situ hybridization (FISH) studies. aCGH testing may be indicated in the following conditions: one or more major anomalies, three or more minor anomalies, unexplained intellectual deficiency, dysmorphism with or without tone and intellectual deficits, unexplained failure to thrive, family history of congenital anomalies, and multiple miscarriages [11]. The FISH test may be used to confirm a microdeletion or microduplication detected by aCGH and is generally used in the prenatal screening of cells in amniotic fluid. In the conditions of consanguinity or suspected uniparental disomy (UPD), where the chromosome material is coming from one parent only, single nucleotide polymorphisms (SNPs) is indicated. Another test, called whole-exome sequencing (WES), is used in children with multiple structural anomalies associated with intellectual disability and/or seizures [12]. This technique uses next-generation sequencing and can simultaneously analyze the coding regions of almost 19,000 to 20,000 genes. While WES cannot be used for the detection of microdeletions or microduplications, whole-genome sequencing (WGS) can detect larger deletions or duplications, as well as triple repeat expansions; and mutations in deep intronic regions, regulatory regions that are outside of the coding regions, and untranslated gene regions. Chromosomal studies should be done in cases suspected of aneuploidy.

The diagnostic tests of mitochondrial diseases include genetic as well as biochemical analyses. Sequencing mtDNA to detect point mutations in muscle, liver, or even urine is generally performed with or without muscle enzymatic studies [13]. Assaying the noninvasive source of mtDNA, such as heteroplasmic load of the common m.3243A > G mutation in uroepithelial cells, is also useful as it closely correlates with the affected tissues in some conditions [14]. Another sequencing technology that has a role in mitochondrial disease evaluation is next-generation sequencing.

Some genetic disorders are metabolic in origin and require metabolic studies in addition to the genetic workup. These studies include tests for blood amino acids and urine organic acids, specific tests for the peroxisomal disorder, including liver biopsy in certain conditions, assessment of serum cholesterol precursors, and lactic acid and pyruvic acid levels in the blood and cerebrospinal fluid, among others. Metabolomics uses tandem mass spectrometry to detect and quantify small molecules in plasma to diagnose inborn errors of metabolism. This procedure scientifically studies the small molecule substrates (<1.5 kDa) called metabolites which are products of the biochemical processes in cellular metabolism. One of the cellular functions that entails mRNA, gene expression, and proteomic analyses leads to the release of gene products in the cell. Metabolomics meets the challenge of systems biology and functional genomics by integrating genomics, transcriptomic and proteomic information. Thus metabolomic information provides a better understanding of cellular biology.

## 9. Risk Assessment of Congenital Anomalies

The establishment of recurrence risks for CA may not always be possible due to the lack of referable evidence. The known recurrence risks of some of the heritable disorders are presented in Table 2.

Some of the risk factors for CA are well documented, such as parental age, subjects’ gender, geographical location and exposure to drugs or toxins, etc. Parental age individually as well as in various combinations has been noted to affect the occurrence of CA in offspring. Advanced maternal age is extensively reported to be associated with aneuploidies, such as trisomy 21, 13, and 18 and Klinefelter syndrome. Advanced maternal age has also been associated with non-chromosomal genitourinary anomalies, as well as hips and feet deformities [17]. Advanced paternal age is associated with an increased incidence of de novo DNA mutations and chromosomal aberrations in the sperm, which may lead to miscarriage or genotypical and/or phenotypical anomalies in the fetus [18]. Like advanced maternal age, trisomy 21 is documented to be associated with advanced paternal age [19]. Other disorders reported to occur more commonly with advanced paternal age are achondroplasia, osteogenesis imperfecta, and some syndromes such as Apert, Waardenburg, Marfan, and Treacher Collins. It had been documented that the risks for cleft palate, diaphragmatic hernia, right ventricular outflow tract obstruction, pulmonary valve stenosis, anomalous pulmonary venous return, cataract, aortic coarctation, encephalocele, esophageal atresia, and multiple complex defects in the offspring are enhanced with each unit year increase in the paternal age [18]. It is noteworthy that the effects of paternal age might vary in tandem with maternal age. While young maternal and paternal age are identified as independent risk factors for gastroschisis, young paternal age can become a risk factor for gastroschisis again if the mother’s age exceeds 35 years. Omphalocele, spina bifida, orofacial clefts, and septal heart defects display associations with parental mating, which involves advanced paternal and young maternal age, and also between a young father and mother of advanced age.

Some diseases exhibit gender preferences [20,21]. The diseases known to occur more commonly in males are pyloric stenosis, Hirschsprung’s disease, imperforate anus, club foot, unilateral multicystic dysplastic kidney; cleft lip and palate, Poland sequence, ventricular septal defect, transposition of great vessels, aortic coarctation, hypoplastic left heart syndrome, subdiaphragmatic total anomalous pulmonary venous return, and pulmonic stenosis and atresia. Disorders identified to be more common in females are choanal atresia, choledochal cyst, congenital hip dysplasia, ureterocele, Trisomy 18, atrial septal defect, patent ductus arteriosus, anencephaly, meningomyelocele and congenital hypothyroidism. In a recent study of 12,795 cases with CA, male fetuses were found to be more susceptible to birth defects than females, however, with significant heterogeneity in the subtypes [20]. Sex organ anomalies are reported to be 8.5 times more common, and gastrointestinal defects 55%, whereas urinary tract anomalies are 62% more prevalent in males than in females. Overall, the prevalence of major CA in males is 3.9% compared to 2.8% in females [21].

## 10. Calculation of Carrier Frequency and Recurrence Risk in a Population

The Hardy–Weinberg equilibrium is utilized to predict carrier frequency in a given population and to calculate recurrence risk. The calculation assumes that the mating is random and there are no new mutations or natural selections.

Calculation of Carrier frequency: Example: normal gene frequency = P; abnormal gene frequency = Q; P + Q = 1 (i.e., 100%); 2PQ = carrier frequency; P^2^ = normal, non-carrier frequency; Q^2^ = affected frequency.

Let us take the example of cystic fibrosis (CF), an autosomal recessive disease. The disease frequency of the morbidity is 1 in 2500 Caucasian births, i.e., Q^2^ = 1/2500; therefore Q = 1/50. We know that P + Q = 1. So P = 1-Q or 1 − 1/50 = 49/50 ~ 1. Therefore 2PQ (carrier frequency) = 2 × 1 × 1/50 = 1/25.

Calculation of recurrence risk of CF: Example: a pregnant woman has a sibling with CF. What are the chances of her having an affected child?

The father’s risk of being a carrier is 1/25, while the mother’s chances of being a carrier are 2/3 as both of her parents are carriers. The chance of the offspring getting the gene from each parent is ¼. Therefore the chances of the child being affected by CF are 1/25 × 2/3 × 1/4 = 1/150.

## 11. Clinical Care and Outcome

Clinical care of CA is highly individualized and guided by the nature, location, and severity of the malformation. Depending upon the defect, it may require a multi-disciplinary approach and involve pediatricians, geneticists, plastic surgeons, neurologists, cardiologists, pediatric surgeons, orthopedics surgeons, dermatologists, infectious disease specialists, and other specialties as indicated. The role of the social worker is vital for family and social-financial support. The natural course, complications, and eventual clinical outcomes depend on the anomaly and may range from inconsequential to severe, and even death. Death from lethal anomalies can occur in utero, at birth, during the perinatal period, or later on in life. The longevity and quality of life depend upon the diagnosis as well as multiple other factors that include constitutional, socio-economical, and even geographical variables. Some patients may have an entirely normal span and quality of life, all determined by the diagnosis.

## 12. Prevention

The prevention of CA depends on the elimination or control of the etiological factors. The methods may be maternal immunization and prophylaxis against known cytopathic viruses, avoidance of cytopathic substances during pregnancy, and application of community genetics. Preconception and antenatal care in terms of maternal nutrients supplementation, treatment of pregnancy associated diseases including infection, and elimination of exposure to illicit and hazardous substances are simple but effective measures. Prevention of genetic disorders with epidemiological data on the prevalence and by evaluating risk assessment in parents with appropriate counseling is of paramount value.

## 13. Summary

Congenital anomalies are a heterogeneous group of disorders of abnormal morphogenesis, which present at birth and carry widely variable implications for morbidity and mortality. They can impose tremendous physical, social, and emotional distress on the patient with massive emotional, social, financial, and medical implications for the family and society. The diagnosis may remain elusive despite rigorous, elaborate, and extensive investigations in many cases. While the enormous strides in genetic testing and gene modification therapy have an encouraging impact on the diagnosis and treatment, the risk assessment of recurrence in the family and population of CA remains obscure in most cases due to the lack of information and referable evidence.

## Figures and Tables

**Table 1:** Common genetic tests for congenital anomalies.

Single Gene Testing	Looks for a Specific Variant in One Gene to Identify Changes That Caused the Anomaly
Targeted gene panel	Tests for variants in more than one gene if anomalies fit a wide array of conditions. Often used to determine familial condition.
Exome sequencing	Looks at all exons, the parts of genes that code for proteins. Compares the parents’ and the affected child’s DNA.
Genome Sequencing	Checks all genetic information to find an explanation for anomalies. Most comprehensive genetic test.
Whole-exome sequencing/whole genome sequencing	Analyzes the bulk of an individual’s DNA. Used when the genetic cause is unclear after preliminary testing. More cost and time effective than performing multiple tests.
Gene expression	Tests for turned off or expressed gene in different types of cells. When a gene is turned on (active), the cell produces mRNA with instructions from the genes. The mRNA molecule is used as a blueprint to make proteins.

**Table 2:** Recurrence risk of certain malformations [15,16].

Disease	Condition	Recurrence Risk
Pyloric stenosis	Mother affected	19% risk for male, 7% for female offspring
	Father affected	5.5% risk for male, 2.4% for female
	1 child affected	4% risk to next male 2.4% to female child
Cleft lip	Unaffected parents, 1 child affected	4.5% risk for next child
	1 parent and 1 child affected	10% risk to next child
Cleft palate	1 child affected	2.6% risk to sibling
Congenital hip dysplasia	1 affected child	0.5% risk for male 6.3% for female child
Cardiac defects	1 child affected	3.4% risk for next child
	2 children affected	10% risk for next child
Neural tube defects	1 child affected	3.5% risk to next child
Trisomy 21	Mother with balanced translocation	10–15% risk for sibling
	Father with balanced translocation	5% risk for sibling
	1 child affected, no parental translocation	1% risk for sibling
Hirschsprung’s disease	1 child affected	3–5% risk for next child
Club foot	1 affected child	2% risk if the 1st child is male, 5% if female
	1 parent and 1 child affected	25% risk to next child
